# A Critical Review on Microalgae-Enhanced Fountain Landscapes for Urban Carbon Capture

**DOI:** 10.3390/microorganisms14061344

**Published:** 2026-06-15

**Authors:** Ling Wang, Mingjing Zhang, Chenba Zhu, Jialin Wang, Chen Hu, Lei Li

**Affiliations:** 1 Technology Innovation Centre for Exploitation of Marine Biological Resources, Third Institute of Oceanography, Ministry of Natural Resources, Xiamen 361005, China; 2 Carbon Neutral Innovation Research Center, Xiamen University, Xiamen 361005, China; 3 College of Ocean and Earth Science, Xiamen University, Xiamen 361005, China; 4 Global Ocean Negative Carbon Emissions (ONCE) Program, Research Center for Ocean Negative Carbon Emissions, Xiamen 361000, China; 5 Fujian Key Laboratory of Marine Carbon Sequestration, Xiamen University, Xiamen 361005, China; 6 Jiangmen Laboratory of Carbon Science and Technology, Jiangmen 529100, China

**Keywords:** microalgae, urban carbon capture, microalgae-based fountain landscapes, photobioreactors, urban sustainability

## Abstract

Achieving carbon-neutral cities requires innovative strategies that integrate technological carbon capture, sustainable urban infrastructure, and proactive public engagement. While microalgae-based systems have shown promise for CO_2_ sequestration and resource recovery, their scalability remains constrained by high costs and energy-intensive photobioreactor (PBR) designs. Here, we propose the retrofit of existing urban fountains into high-efficiency microalgae cultivation systems—microalgae-enhanced fountain landscapes—as an integrated solution that bridges ecological function and social outreach. This approach capitalizes on ubiquitous fountain infrastructure to minimize deployment costs, employs advanced fountain-style cultivation technology to enhance biomass productivity, and leverages strategic locations in high-footfall urban zones to actively elevate public carbon literacy and motivate low-carbon behavioral shifts through immersive engagement—a vital step toward city-wide participatory climate action. We critically analyze the feasibility of this system, highlighting its potential for multi-stakeholder value creation across developers, municipalities, and citizens. Furthermore, we synthesize recent advances in suspended microalgae cultivation, building-integrated PBRs, and microalgae-informed landscape design to contextualize the development pathway of fountain-based systems. By uniting technical efficiency with civic education, this work establishes a replicable framework for scalable urban deployment—simultaneously advancing carbon mitigation, public awareness, and circular resource flows in the transition toward climate-resilient cities.

## 1. Introduction

The escalating atmospheric carbon dioxide (CO_2_) concentrations are intensifying global environmental crises, including global warming and ocean acidification [1]. Consequently, achieving the Paris Agreement’s 1.5 °C target necessitates urgent and ambitious carbon neutrality strategies, a commitment many nations and institutions have embraced for the coming decades [2,3]. However, such actions inherently conflict with economic development, evidenced by projections that China would incur gross domestic product (GDP) losses of 2.8–5.7% to meet 1.5 °C goals by 2050 [4,5]. Consequently, technologies capable of simultaneous CO_2_ sequestration and economic growth are critical for viable decarbonization pathways.

These imperatives critically intersect with intensifying global urbanization. As urban populations expand, environmental pressures escalate, manifesting in compromised water quality from inadequate waste management, severe ambient air pollution, and diminished green infrastructure [6,7,8]. Critically, high-density urbanization acts as a primary accelerator of carbon emissions and systemic ecological degradation: urban centers occupy merely 3% of global land yet account for approximately 80% of total greenhouse gas emissions [9]. Thus, urban densification disproportionately concentrates ecological burdens, acting as key multipliers of anthropogenic climate disruption and demanding integrated mitigation strategies central to carbon neutrality efforts.

Alongside systemic and technological decarbonization, active citizen engagement constitutes an essential dimension for achieving carbon neutrality. While industrial fossil fuel combustion dominates global emissions, human behavior remains the fundamental driver, in which individual actions offer critical leverage points for emission reduction [10]. Consequently, widespread adoption of sustainable practices is imperative, where promoting green and low-carbon lifestyles across populations holds substantial mitigation potential. For instance, behavioral interventions could reduce global carbon emissions by 19.9–36.8% between 2020 and 2050 [11]. Hence, alongside developing sequestration technologies and cultivating technical expertise, strategic public education and engagement in low-carbon living and production patterns are paramount for achieving viable carbon neutrality.

**Figure 1:**
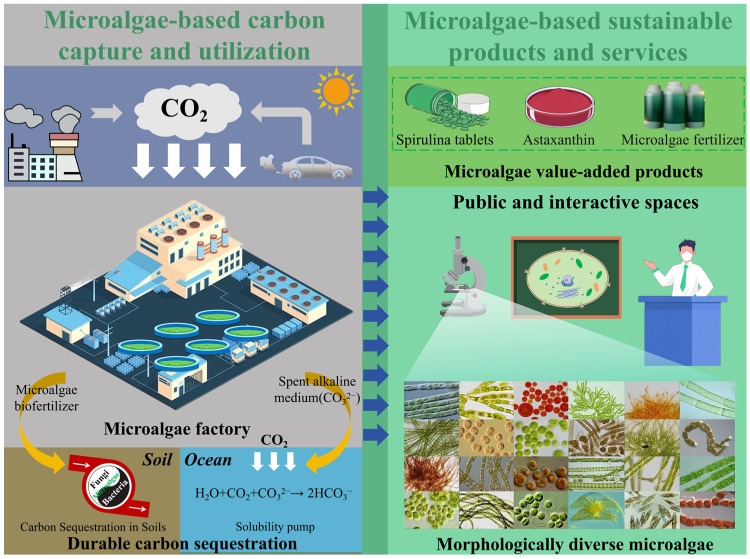
Microalgae-based carbon capture and utilization technology for sustainable development.

Photosynthetic microalgae represent a promising candidate for CO_2_ sequestration and renewable energy production, exhibiting rapid growth rates, high photosynthetic efficiency, substantial CO_2_ fixation capacity, and non-competition with arable land [12]. Significantly, microalgae demonstrate dual environmental utility through (Figure 1) (1) wastewater remediation via efficient assimilation of nitrogen and phosphorus [13,14,15,16] and (2) flue gas bio-fixation by sequestering CO_2_ [17], thereby transforming pollutants into biomass. Furthermore, their biomass accumulates high-value compounds (pigments, proteins, lipids), providing sustainable and circular economic pathways [18]. Consequently, microalgae-based technology enables sustainable deployment of urban cities by simultaneous carbon sequestration, resource recovery, and economic growth (Figure 1). However, current microalgal commercial production exhibits inherent limitations including suboptimal productivity, elevated operational expenditures, and intensive energy demand [19,20,21,22], hindering large-scale deployment.

**Figure 2:**
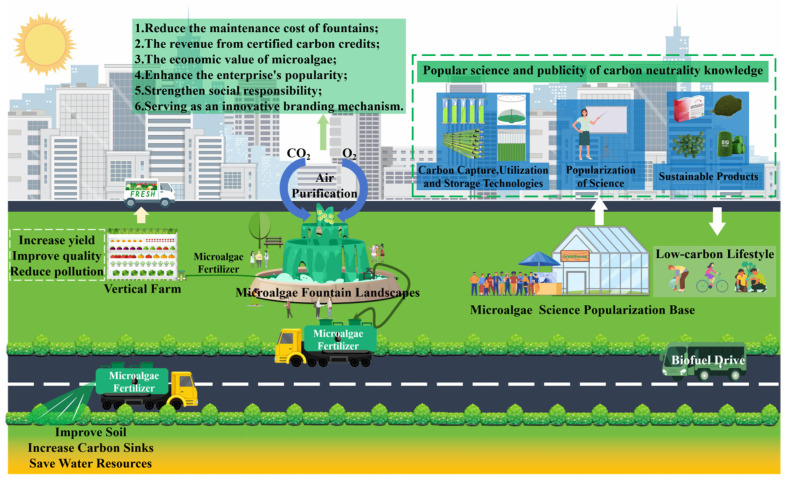
Schematic of a microalgae-enhanced fountain landscape for integrated carbon capture technology and carbon neutrality public education.

While microalgae-based carbon sequestration currently focuses predominantly on biomass carbon fixation and faces economic/energy constraints, the unique properties of microalgae offer unexplored pathways for public engagement. For instance, microalgae-derived high-value nutraceuticals and their distinctive and ravishing structural/color properties possess inherent market appeal and established commercial presence (Figure 1). These attributes position microalgae as compelling vectors for carbon neutrality education; however, this opportunity is largely overlooked in current strategies. Herein, this study proposes deploying microalgae-enhanced fountain landscapes in urban cities as integrated systems achieving dual objectives (Figure 2): (1) environmental remediation through on-site CO_2_ bio-fixation, air/water purification, renewable energy production and resource recovery; (2) public engagement by creating visually striking installations in high-footfall areas that attract citizens. Such installations transform passive infrastructure into active educational platforms, leveraging aesthetic appeal to disseminate knowledge on circular bioeconomy principles and carbon neutrality for promotion of low-carbon lifestyles in citizens.

In this study, the deployment framework for the proposed microalgae-enhanced fountain landscapes is critically analyzed and discussed, with a focus on multi-stakeholder value generation among developers, infrastructure owners, municipal authorities, and citizens. To contextualize this innovation, we synthesize advancements across three domains: (1) suspended microalgae cultivation systems, (2) building-integrated photobioreactor (PBR) applications, and (3) microalgae-based landscapes. Building on this foundation, future development pathways are outlined to optimize the efficiency, scalability, and sustainability of microalgae fountain implementations. This study provides an innovation and practical strategy for the achieving goals of carbon neutrality and the sustainable development of urban cities, demonstrating strong potential for scalable implementation to advance climate-resilient urbanization.

## 2. Progress on Suspended Microalgae Cultivation Systems for Carbon Capture and Fixation

Microalgal carbon capture and fixation begin with carbon supply and capture, which can proceed through multiple routes based on the different forms of carbon source. The first route uses gaseous CO_2_: CO_2_-enriched or ambient air is sparged into the medium, and the resulting dissolved CO_2_ is directly taken up for photosynthesis [12,17]. The second route relies on endogenously generated bicarbonate (HCO_3_^−^): the dissolved CO_2_ can be further converted into HCO_3_^−^/CO_3_^2−^ based on carbonate chemical equilibrium; microalgae then take up HCO_3_^−^ via diffusion or HCO_3_^−^ transporters and transport it into the cell, where intracellular carbonic anhydrase releases CO_2_ from HCO_3_^−^, and elevate the CO_2_ concentration around Rubisco [12,19]. This process is named the carbon-concentrating mechanism (CCM). The third route directly supplies HCO_3_^−^ as an external carbon source, exploiting the same CCM machinery for carbon capture [23,24].

The captured carbon is subsequently fixed and assimilated as organic biomass components such as carbohydrates and proteins through the Calvin–Benson cycle, thereby converting inorganic carbon into harvestable biogenic carbon [18,20]. Notably, for capture routes 2 and 3 that rely on HCO_3_^−^, the spent medium is enriched with CO_3_^2−^ and has a high pH. Consequently, this alkaline spent medium can be re-gassed with CO_2_ to enable another capture-fixation cycle, improving carbon utilization efficiency, and supports closed-loop carbon capture and reuse systems [19,25]. In engineered systems, the net carbon capture-fixation outcome further depends on several factors such as light distribution, mixing efficiency, pH, cultivation system, and algal strain. Table 1 accordingly focuses on representative cultivation datasets and compares how different carbon sources and culture conditions influence biomass output and CO_2_ fixation performance.

**Figure 3:**
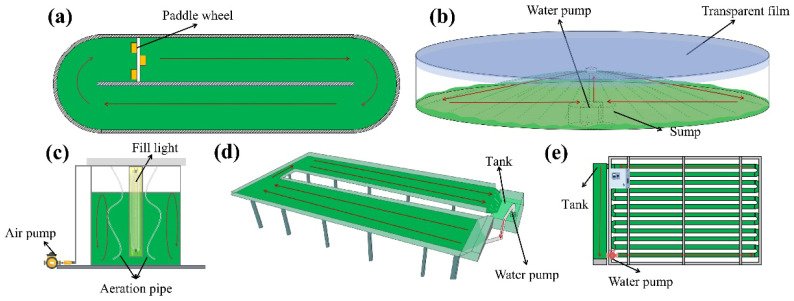
Diagram of suspended microalgae cultivation systems: (**a**) raceway pond; (**b**) thin-layer fountain photobioreactor (PBR); (**c**) flat-panel PBR; (**d**) thin-layer cascade system; (**e**) tubular PBR. Note: The red arrows in the figure indicate the trajectories of the microalgal solution movement.

Microalgae production systems constitute the technological foundation of microalgae-enabled carbon neutrality strategies [19], since they directly determine cultivation economics, energy consumption, and operational scalability. The two mainstream cultivation paradigms are biofilm-based attached systems and suspension-based liquid-medium systems. Although the attached systems exhibit benefits in harvesting efficiency, photosynthetic productivity, and water conservation [32], their large-scale application is hindered by inherent limitations such as restricted light penetration, inefficient CO_2_ diffusion, high energy inputs, and demanding maintenance [33]. Conversely, suspended cultivation systems, exemplified by open ponds and tubular photobioreactors (PBRs) (Figure 3), have been widely commercialized at industrial scale owing to their proven scalability and operational maturity [34,35]. Given these considerations, this paper focuses specifically on suspended microalgae cultivation.

Nevertheless, suspended systems face distinct challenges. Open raceway ponds, for instance, are prone to contamination, exhibit limited areal productivity, suffer from substantial water evaporation, and require large land footprints [36,37,38]. Enclosed photobioreactors (e.g., tubular and flat panel designs) provide superior regulation of cultivation conditions and achieve greater output efficiency [39]. Nevertheless, their widespread adoption is primarily restricted to the production of premium compounds, exemplified by astaxanthin derived from *Haematococcus pluvialis*, owing to substantial capital investment, intricate operational demands, and challenges in scale-up [40].

A common technical limitation underlying the high microalgal biomass production costs of both systems is their high culture depth (typically 5–30 cm) [41]. This design leads to rapid light attenuation and significant mutual shading among cells, which severely inhibits photosynthetic efficiency. As a result, cell densities remain below 2.0 g·L^−1^ [42,43], and areal productivity rarely exceeds 15 g·m^−2^·d^−1^. The low biomass concentration not only constrains overall output but also increases downstream processing costs, in which harvesting process alone can account for over 30% of the total expense [44]. Consequently, even for relatively low-cost strains such as *Spirulina* sp., production costs typically exceed $3.0/kg [45,46], severely restricting industrial scalability. For instance, China, despite being the world’s leading microalgae producer, possesses an annual dried biomass production capacity of merely around 10,000 tons [47]. This modest output significantly constrains its viability for extensive carbon sequestration initiatives.

In recent decades, thin-layer cascade systems (TLCS) emerged as a groundbreaking methodology, specifically designed to address the inherent limitations of conventional deep-culture systems [48,49,50]. Within the TLCS, microalgae culture is pumped from a retention tank to the apex of an inclined channel. From there, it passively returns to the tank via gravity, completing the continuous recirculation loop. This novel design enables ultralow culture depths (<1 cm) [51,52], yielding high cell densities (50.0 g·L^−1^) and biomass productivities (15–50 g·m^−2^·d^−1^). However, their rigid-material construction generates prohibitively high capital investment, offsetting the operational advantages and perpetuating the economic constraints of commercial microalgal production.

Addressing these limitations, a novel thin-layer fountain photobioreactor (TLF-PBR) employing water-pump-driven spray circulation was developed to achieve layer depth control (1.0–3.0 cm) [53]. Operating at 1.0–3.0 cm depths, a 1.5 m diameter TLF-PBR achieved 4.86 g·L^−1^ biomass density, a 44.7% increase over conventional TLCS. Zhang et al. [54] further enhanced performance through variable-frequency mixing technology (VFMT), elevating biomass productivity by 9.34–40.93% while reducing mixing energy consumption by 27.73%. Crucially, VFMT achieved superior mixing energy utilization efficiency (100–250 g·kWh^−1^). These advancements position TLF-PBRs as scalable platforms adept at harmonizing operational efficiency and economic feasibility for both microalgal biosynthesis and carbon dioxide sequestration.

## 3. Progress on Microalgae-Based Buildings and Landscapes

The unique biological functions of microalgae, including CO_2_ sequestration, oxygen release, and temperature and humidity regulation, enable their deep integration into buildings and landscapes [55,56]. This multifunctional living system could simultaneously purify indoor air, modulate thermal–humidity conditions, and generate biomass for onsite bioenergy production to supply heating systems. Consequently, microalgae-based architecture has evolved into a rapidly advancing multidisciplinary paradigm that combines biotechnology with sustainable building and landscape design [57,58,59,60]. By transforming buildings and landscapes into photosynthetic powerhouses, this approach creates regenerative ecosystems that enable synergistic circularity through simultaneous energy harvesting, carbon cycling, and climate regulation, offering a promising pathway for sustainable urban development.

### 3.1. Microalgae-Based Buildings and Their Development

**Figure 4:**
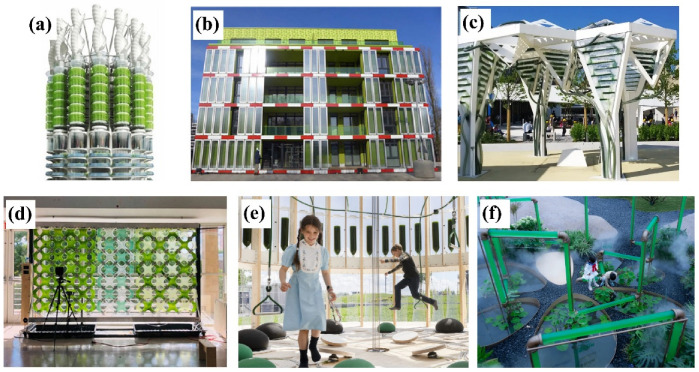
Microalgae-based building and landscape: (**a**) Algae Green Loop on the marina city towers; (**b**) Bio-Intelligent quotient building (BIQ); (**c**) urban algae canopy; (**d**) microalgae windows; (**e**) air bubble playground; (**f**) Greenwich microalgae experimental garden.

Due to the above significant advantages, microalgae-based buildings have been widely proposed and developed in recent decades. For instance, the Algae Green Loop (Figure 4a) represents the first architectural proposal integrating on-site PBRs with direct air capture technology for carbon sequestration. This project established a closed-loop sustainability model targeting urban carbon neutrality through its implementation at Chicago’s Marina City Towers, where helical PBRs envelop the tower perimeters. The system achieved triple carbon reduction via (1) atmospheric CO_2_ capture using humidity-swing adsorption; (2) photosynthetic sequestration by microalgae; and (3) renewable energy substitution (solar/wind). Critically, the design institutes a participatory circular economy through an eco-market bridge, enabling residents to exchange vertically farmed produce within an algae-integrated showroom. This intervention transforms urban dwellers into stakeholders in the building’s metabolic cycle.

Germany spearheaded a global first in 2013 with the inauguration of the Powerhouse (BIQ) in Hamburg (Figure 4b), an algae-powered building incorporating 129 facade-integrated flat-panel PBRs [61]. These PBRs harness photosynthetic processes to simultaneously generate thermal energy and microalgal biomass for biofuel production, meeting 30% of the building’s total energy demand. Critically, this project established the first operational integration of microalgal cultivation, renewable energy production, and architectural infrastructure at scale, demonstrating onsite symbiosis between biological systems and building environments.

Advancing this paradigm, EcoLogic Studio unveiled the Urban Algae Canopy (Figure 4c) at the Venice Architecture Biennale in 2015 [62], which is made from three layers of ethylene tetrafluoroethylene membrane (ETFE) cladding. It employed computer-numerically controlled fabrication to design the form of the cushions and to control the fluid dynamic behavior of the water medium as microalgae travels through it. This innovative structure integrates ETFE membranes with PBRs to concurrently provide solar shading, atmospheric purification, and biomass production.

PBRs can also be architecturally integrated as algae-infused windows (Figure 4d) to enhance energy efficiency and environmental performance [63]. This integration delivers multifaceted benefits, including energy conservation, optimized daylighting, hot water production, and carbon sequestration [64,65]. Demonstrating the potential of such integrated designs, Kim et al. [66] engineered a microalgae–PBR window system utilizing *Chlorella* sp. and *Chlorococcum* sp. This system achieved daily biomass productivities of 175 mg·L^−1^·d^−1^ and 80 mg·L^−1^·d^−1^, respectively, offering compelling evidence for the feasibility of microalgal building facades in active carbon capture applications.

### 3.2. Microalgae-Based Landscapes and Their Development

Additionally, microalgae biotechnology has expanded into urban landscape design through biologically integrated systems. Microalgal landscapes constitute functional biological modules or networks thereof [67], which merge microalgal biotechnology, ecological aesthetics, and societal needs within urban contexts [68]. These units provide habitat provisioning and ecosystem services while establishing interconnected metabolic networks that enhance urban sustainability. This framework fosters mutualistic symbiosis between humans and microorganisms, offering a novel pathway for sustainable urban transition and climate resilience.

In 2021, London-based ecoLogicStudio commissioned the world’s first air bubble playground (Figure 4e) in central Warsaw [69], integrating microalgal air-purification biotechnology within recreational infrastructure. The cylindrical structure employs timber frames and ETFE membranes housing 52 tubular PBRs. These PBRs feature interactive elements (foot pumps, tension ropes, rebound spheres) serving dual functional purposes while providing 200 L·min^−1^ collective air filtration capacity. The symbiotic system operates through dual energy inputs: solar radiation powers photosynthesis while children’s kinetic engagement drives air circulation through the PBRs. This establishes a novel child–microbe cohabitation model where recreational behavior synergizes with algal metabolism to enhance climate adaptation and pediatric respiratory health through integrated air purification and microclimate regulation [70].

In 2024, Aobo Landscape Design Co., Ltd. established China’s inaugural microalgae park (Greenwich microalgae experimental garden, Figure 4f) in Chengdu, integrating tubular PBRs as biocultural components to create an organically unified environmental system. This carbon-neutral demonstration garden generates vibrant urban green space while facilitating public understanding of microalgal ecology, growth parameters, and carbon-neutral technologies. To enhance visitor engagement, an interactive motion-tracking installation at the entrance stimulates visitor exploration by dynamically responding to movement. Complementing this intervention, fog/mist systems maintain optimal humidity for floral cultivation while producing soft veiling effects that create ethereal visual experiences through atmospheric light diffraction. These integrated features collectively transform the space into an immersive environmental education platform where aesthetic immersion facilitates experiential learning about photosynthetic carbon sequestration pathways.

## 4. Development and Feasibility of Microalgae-Enhanced Fountain Landscapes

Despite over a decade of development, microalgae-integrated architecture remains commercially unrealized at scale due to prohibitive capital/operational expenditures for PBR fabrication, deployment, and maintenance [39,71,72]. These economic constraints intensify when systems are installed on building envelopes, where installation complexity compounds maintenance challenges [73,74]. Furthermore, predominantly decentralized small-scale deployment yields insufficient biomass volumes to achieve economies of scale, critically impeding commercial viability.

To overcome commercialization barriers in microalgae-integrated architecture, this study proposes retrofitting existing fountain infrastructure with high-efficiency cultivation technology to create microalgae-enhanced fountain landscapes (Figure 2). This approach achieves dual objectives of productive biomass generation and sustainable landscaping while circumventing prohibitive capital/operational expenditures through structural repurposing. The fountain-based cultivation technology enables high-density cultivation (>5 g·L^−1^) with enhanced biomass productivity and reduced energy inputs [53]. This integrated solution provides cities with a technically and economically viable pathway for low-carbon transition, simultaneously demonstrating carbon sequestration through visually demonstrative systems while creating multifunctional public spaces that enhance urban livability and climate resilience.

Beyond their role in carbon sequestration and environmental improvement, microalgae-enhanced fountain systems serve as distinctive and effective platforms for public carbon neutrality education, leveraging synergies such as the widespread presence of existing fountain landscapes that naturally attract visitors in urban recreational areas, along with the inherent appeal of microalgae evidenced by their vibrant colors, diverse forms, and documented health benefits [75,76,77]. By integrating carbon literacy stations into these engaging settings, the systems can effectively communicate the principles and technologies of carbon neutrality while inspiring meaningful shifts in public behavior. Importantly, this approach extends climate impact beyond biological carbon capture by raising awareness and motivating the adoption of low-carbon lifestyles in citizens, thereby activating participatory emission reductions that are essential to achieving carbon neutrality goals [10].

The development and implementation of microalgae fountain landscapes deliver multi-stakeholder benefits extending beyond technology developers, the public and policymakers to fountain owners. First, high-density microalgal cultivation within these systems mitigates eutrophication and microbial bloom risks inherent in conventional fountains, significantly reducing maintenance costs for owners. Second, the captured CO_2_ emissions from fountain pump operations enable microalgae growth, yielding certified carbon credits (CERs) that generate ancillary revenue streams. Concurrently, the harvested microalgae biomass provides feedstock for value-added commercial applications. Third, integrated carbon literacy platforms disseminate carbon neutrality technologies while showcasing corporate sustainability initiatives. This dual function enhances corporate visibility and strengthens perceptions of social responsibility, serving as an innovative branding mechanism. Thus, by delivering environmental remediation, economic returns, and societal engagement, microalgae-enhanced fountain landscapes present a compelling multi-stakeholder value proposition conducive to widespread application in future.

## 5. Challenges and Prospects

Although microalgae-enhanced fountain landscapes hold considerable promise for urban carbon capture and sustainability communication, their large-scale deployment remains constrained by several unresolved challenges. These include limited cultivation efficiency, an uncertain net carbon balance, difficulties in decentralized biomass valorization, and the need for sustained public participation.

To address these barriers, future research and demonstration projects should focus on three interconnected priorities: improving cultivation and energy performance, developing practical low-energy biomass utilization pathways, and strengthening the long-term educational and social functions of these systems.

(1) To further elevate carbon sequestration efficiency and biomass productivity in microalgae-enhanced fountain systems, future research must prioritize optimization of hydrodynamic mixing and energy-efficient cultivation processes. Mixing intensity represents a critical determinant of microalgal growth kinetics, which regulate mass transfer gradients, thermal homogeneity, and photocycle frequency to govern photosynthetic efficiency and carbon fixation rates [78,79]. However, current mechanical mixing contributes 22–79% of total energy consumption in microalgae cultivation stage [80], potentially transforming these carbon-capturing systems into net carbon sources when powered by fossil fuels [81].

To mitigate this paradox, integrating renewable energy-driven mixing technologies was proposed, particularly photovoltaic-powered fluidic systems. Such hybridization would eliminate operational carbon emissions while enhancing net sequestration potential. Computational fluid dynamics modeling should be employed to establish genotype-specific mixing parameters that maximize mixing efficiency without compromising cellular integrity. Concurrently, life-cycle assessments must quantify the carbon breakeven thresholds under different energy scenarios, ensuring the technology delivers verifiable climate-positive outcomes.

(2) While microalgae-integrated fountain systems demonstrate significant promise, their broad implementation is hampered by economic barriers. These constraints arise from scattered deployment scenarios and the considerable expenses involved in microalgal harvesting, dewatering, and drying processes [82,83]. To address this challenge, we propose to utilize unharvested microalgal culture broth as a direct-use liquid biofertilizer in future. This strategy eliminates energy-intensive processing while leveraging the proven benefits of microalgal fertilizers [84,85,86,87], which include enhancing soil health, improving crop yield, partially replacing synthetic fertilizers, promoting soil carbon sequestration, and supporting sustainable food production. Crucially, the liquid application aligns with the decentralized nature of fountain systems, and facilitates dual urban resource loops: supplying nutrient-rich cultivation media to urban vertical farms for local sustainable food production, while also serving as irrigation water for urban greening (Figure 2). This integrated strategy not only alleviates water management constraints but also enhances carbon sink capacity through soil amendment, demonstrating a circular economy model that advances both carbon neutrality and sustainable urban agriculture for scalable implementation.

To maximize these agronomic and climate benefits, future research must prioritize mechanistic investigations into rhizosphere processes governing crop productivity, particularly focusing on nutrient solubilization dynamics, phytohormone modulation, and microbiome recruitment. Concurrently, fundamental studies should elucidate carbon sequestration mechanisms (e.g., mineral-associated organic matter formation, microbial carbon pump efficiency) in fertilized soils [88,89]. These insights should directly inform precision formulation technologies optimizing microalgal strain selection, cultivation regimes, and targeted bioaugmentation strategies to enhance site-specific soil–carbon dynamics.

(3) To significantly elevate public engagement and broaden the appeal of microalgal fountain landscapes as sustainable urban features, a multi-pronged strategy is proposed. First, integrating synchronized light and music performances, potentially featuring renowned environmentally conscious musicians, can dramatically increase visibility and cultural resonance, transforming the installation into a compelling artistic and ecological attraction. Second, embedding interactive technology, such as voice-responsive systems allowing public participants to modulate fountain dynamics (e.g., flow frequency and height), will foster direct experiential learning and a tangible sense of agency. Complementing this interactivity, the issuance of verifiable participation certificates quantifying individual contributions to carbon sequestration metrics reinforces the direct link between public action and environmental benefit, explicitly highlighting the system’s roles in atmospheric CO_2_ capture and renewable biomass production.

Additionally, the distribution of complimentary samples of microalgae-derived sustainable and low-carbon products such as biofertilizers serves as a critical link between public awareness and tangible sustainable consumption. This approach not only demonstrates the practical outputs of the circular bioeconomy in an accessible manner but also educates the public on how carbon-captured biomass can be transformed into daily-use goods. By physically connecting the fountain system’s ecological function to real-life applications, it strengthens public recognition of microalgae’s value beyond aesthetics, fostering a deeper understanding of sustainable production and incentivizing pro-environmental consumer behavior.

Together, these integrated strategies—combining cultural appeal through performances, hands-on interactive technology, quantifiable environmental recognition, and product-based learning—collectively transform the fountain system into a powerful carbon neutrality communication platform. They are designed not only to engage a broad urban audience but also to intuitively convey the principles of carbon capture, circular economy, and low-carbon living. By creating an immersive and memorable experience, the platform effectively stimulates lasting awareness and encourages the adoption of sustainable lifestyles, thereby amplifying its overall contribution to urban climate mitigation efforts.

## 6. Conclusions

In summary, retrofitting existing fountains into microalgae-enhanced cultivation landscapes offers a practical strategy to couple CO_2_ capture, biomass generation, landscape renewal, and carbon-neutrality education within the same urban infrastructure. By reusing established fountain assets and integrating high-efficiency shallow-layer cultivation, this concept lowers deployment barriers compared to standalone microalgal systems while creating visible and publicly accessible carbon mitigation demonstrations. With continued optimization in energy use, biomass valorization, and public engagement, this approach holds strong potential to support climate-resilient and low-carbon urban development.

## Figures and Tables

**Table 1:** Representative datasets showing the effects of carbon-source type and culture conditions on microalgal biomass and CO_2_ fixation.

Carbon Source	Culture Conditions	Core Data	Ref.
Simulated waste gas (15% CO_2_)	*Chlorella* sp.; reactor: 5 L PET bottles, closed system; gas: simulated waste gas (15% CO_2_), 0.01 vvm; light: fluorescent light	Biomass productivity: 0.81 g·L^−1^·d^−1^; CO_2_ fixation efficiency: 0.90 g·L^−1^·d^−1^	[26]
Combustion flue gas (15% CO_2_)	*Chlorella* sp.; 1 L bubble column PBR; gas: combustion flue gas (15% CO_2_)	Biomass productivity: 0.192 g·L^−1^·d^−1^ CO_2_ fixation: 0.353 g·L^−1^·d^−1^	[27]
Direct NaHCO_3_ (4.2 g·L^−1^)	*Scenedesmus acuminatus* TH04; reactor: bicarbonate-based reactor; pH: 9.0; gas: bicarbonate supply; light: 250 μmol·m^−2^·s^−1^	Biomass concentration: 1.7 g·L^−1^; HCO_3_^−^ fixation efficiency: 100%	[28]
5% CO_2_ + Na_2_CO_3_/TETA	*S. acuminatus* TH04; reactor: single PBR unit; gas: 5% CO_2_ at 0.1 vvm with 50 mM Na_2_CO_3_ and 5 mM TETA	Biomass concentration: 4.7 g·L^−1^; carbon fixation efficiency: 9.8%	[28]
0.5 g·L^−1^ NaHCO_3_ + 1% CO_2_	*Chlorella* sp. HS2; reactor: shake flask; gas: 1% CO_2_ at 0.25 vvm with 0.5 g·L^−1^ NaHCO_3_	Biomass productivity: 542.9 mg·L^−1^·d^−1^; biomass concentration: NR; CO_2_ fixation efficiency/alternative metric: NR	[29]
pH-stat bicarbonate fed-batch	*Chlamydomonas* sp. JSC4; reactor: fed-batch system; gas: 100 mM bicarbonate feeding	biomass concentration: 1.45 ± 0.32 g·L^−1^; carbon fixation efficiency: 63.8 ± 3.23%	[23]
Absorption-derived KHCO_3_ (5 g·L^−1^)	*Chlorella sorokiniana* SU-1; pH: 8.5; gas: KHCO_3_ derived from flue-gas absorption	Biomass concentration: 1.022 g·L^−1^; carbon utilization efficiency: 86.9%	[24]
Recycled bicarbonate medium (0.3 mol·L^−1^)	*Neochloris oleoabundans* UTEX1185; reactor: 3 L Erlenmeyer flask (0.8 L working volume); pH: 8.5 after CO_2_ replenishment; gas: pure CO_2_ for medium regeneration, no air bubbling during culture; light: 141.5 μmol·m^−2^·s^−1^	Biomass productivity: 0.39 g·L^−1^·d^−1^; carbon utilization efficiency: 98 ± 0.78%	[30]
Air-capture bicarbonate pool (300 mmol·L^−1^)	*Spirulina* sp. DUT001; reactor: 5 L bubble-column PBR; pH: 10.0; gas: air with 300 mmol·L^−1^ bicarbonate/carbonate pool; light: 188.7 μmol·m^−2^·s^−1^	Biomass productivity: 0.575 g·L^−1^·d^−1^; biomass concentration: 3.45 ± 0.08 g·L^−1^; carbon fixation efficiency: 107.5 ± 0.41%	[31]

## Data Availability

The original contributions presented in this study are included in the article. Further inquiries can be directed to the corresponding authors.

## Abbreviations

The following abbreviations are used in this manuscript:

BIQ	Bio-Intelligent quotient
CO_2_	Carbon dioxide
CERs	Certified carbon credits
ETFE	Ethylene tetrafluoroethylene membranes
GDP	Gross Domestic Product
PBR	Photobioreactor
TETA	Triethylenetetramine
TLCS	Thin-layer cascade systems
TLF-PBR	Thin-layer fountain photobioreactor
VFMT	Variable-frequency mixing technology
